# Assessing Fitness-To-Drive among Older Drivers: A Comparative Analysis of Potential Alternatives to on-Road Driving Test

**DOI:** 10.3390/ijerph17238886

**Published:** 2020-11-29

**Authors:** Yongjun Shen, Onaira Zahoor, Xu Tan, Muhammad Usama, Tom Brijs

**Affiliations:** 1 School of Transportation, Southeast University, Nanjing 211189, China; onairakhattak@yahoo.com (O.Z.); 220193153@seu.edu.cn (X.T.); usama@seu.edu.cn (M.U.); 2 Transportation Research Institute (IMOB), Hasselt University, 3500 Hasselt, Belgium; tom.brijs@uhasselt.be

**Keywords:** road traffic safety, older drivers, fitness-to-drive, simulated driving, gradient boosted machine (GBM)

## Abstract

To enable older drivers to maintain mobility without endangering public safety, it is necessary to develop more effective means of assessing their fitness-to-drive as alternatives to an on-road driving test. In this study, a functional ability test, simulated driving test, and on-road driving test were carried out for 136 older drivers. Influencing factors related to fitness-to-drive were selected based on the correlation between the outcome measure of each test and the pass/fail outcome of the on-road driving test. Four potential alternatives combining different tests were considered and three modeling techniques were compared when constructing the fitness-to-drive assessment model for the elderly. As a result, 92 participants completed all of the tests, of which 61 passed the on-road driving test and the remaining 31 failed. A total of seven influencing factors from all types of tests were selected. The best model was trained by the technique of gradient boosted machine using all of the seven factors, generating the highest accuracy of 92.8%, with sensitivity of 0.94 and specificity of 0.90. The proposed fitness-to-drive assessment method is considered an effective alternative to the on-road driving test, and the results offer a valuable reference for those unfit-to-drive older drivers to either adjust their driving behavior or cease driving.

## 1. Introduction

The independence, mobility, and freedom provided by driving are highly valued by the elderly. In recent decades, the number of older drivers has increased due to the aging of the population. The involvement of older drivers in crashes has also increased. Statistics show that older drivers are over represented in fatal and serious injury crash involvements [1,2]. Declining driving ability, combined with increased frailty associated with age, are the major causes of the risk, which can have a severe negative impact on road traffic safety [3]. Therefore, it is of great significance to assess the fitness-to-drive of older drivers and to recognize those who are unfit-to-drive. The on-road driving test is generally regarded as the “gold standard” [4,5], but usually has considerable costs in terms of material, manpower, and time [6], and has potential danger for older drivers with impaired driving ability. As a result, identifying alternatives to the on-road driving test to assess older drivers’ fitness-to-drive is an increasing concern.

In reality, the risk of driving at an older age is not about age itself, but about the physical changes due to aging [7]. The degree of functional deterioration determines whether the elderly can continue driving safely to a large extent [8]. The functional ability usually considered for older drivers includes three main aspects: visual function, motor function, and cognitive function. Visual impairment is the most common sensory problem affecting the driving ability of the elderly. Generally, the elderly suffer from decreased visual acuity and contrast sensitivity, and are more vulnerable to glare [9]. In terms of motor function, older drivers’ reaction and movement times are generally longer than that of younger drivers, which results in reduced driving operation ability [10]. Regarding cognitive function, drivers’ attention, memory, and executive function generally decline with age, which makes it difficult for the elderly to deal with complex driving environments and emergencies safely [3]. Consequently, functional ability is particularly critical to safe driving, and should be an essential part of a fitness-to-drive assessment [11].

A series of fitness-to-drive assessment methods based exclusively on a functional ability test have been established over in recent decades [12,13,14,15,16,17,18,19]. Although different test batteries are applied, few of these achieve the desired accuracy, which indicates the difficulty in discriminating safe and unsafe drivers using a functional ability test alone [11]. The emergence and maturity of driving simulation technology provides a new choice to improve the accuracy of older drivers’ fitness-to-drive assessments. Simulated driving experiments allow researchers to study complex driving behaviors in a controlled environment, which might be impractical, unsafe, or unethical under actual road conditions [20]. By comparing the performance of naturalistic driving and simulated driving of the elderly, several studies have supported the effectiveness of applying a driving simulator to measure driving behavior [6,21]. These experiments can not only collect longitudinal and lateral driving performance parameters of older drivers during normal driving, but they can also record their performance in specific events that are known to be difficult to execute, such as yielding at an intersection, turning left, and merging into traffic [4,11,19]. Previous studies established a fitness-to-drive assessment method by combining a neuropsychological test, clinical interviews, and simulated driving, with accuracy of 93% for patients with dementia [4] and 94.4% for patients with mild cognitive impairment (MCI) [22]. The value of well-designed simulated driving experiments to study the driving safety of the elderly is considerable.

Although the abovementioned studies have combined a variety of assessments with simulated driving tests and obtained good results, concerns remain. First, the subjects of these studies were specific populations with either Alzheimer’s disease or dementia, so the assessment of their performance in the simulator was relatively simple. Moreover, most studies applied traditional statistical models, such as a regression model, to distinguish between safe and unsafe drivers, but few took model generalization into account. In addition, to find an effective alternative to the on-road driving test for assessing fitness-to-drive among older drivers, the balance between the achievable assessment performance and the potential costs, including labor, raw material, and time should be taken into consideration. As a result, the fitness-to-drive assessment of older drivers with more general characteristics is investigated in this study. The study considered two simulated driving assessment methods, i.e., observation-based and performance-based and investigated three advanced data mining and machine learning techniques, i.e., random forest (RF), support vector machine (SVM), and gradient boosted machine (GBM). Finally, four potential alternatives to the on-road driving test, based on either the functional ability test alone or different driving performances in simulators, or their combination, were compared.

The remainder of this paper is organized as follows. The experiments carried out and the data collected for the study are described in Section 2. Next, methodology related to variable selection and model development are specified in Section 3. The results from the models are presented in Section 4 and further discussed in Section 5. The paper ends with conclusions and future research in Section 6.

## 2. Data Description

Participants were recruited through the geriatrics day hospital of the Jessa Hospital (Belgium), in addition to information brochures issued by local elderly organizations. Active drivers over 70 years old who presented or were suspected to have cognitive decline were included, and were informed of the procedure of the study. The participants had their functional ability test in Jessa Hospital on day one, a driving simulator ride with a trained researcher from the Transportation Research Institute (IMOB) of Hasselt University on day two, and an on-road driving test similar to the Belgian official licensing procedure on day three.

In total, 136 older drivers (102 males and 34 females) agreed to participate in the study and provided written informed consent. This study protocol was approved by the ethical review committees of Hasselt University and the Jessa Hospital.

### 2.1. Functional Ability Test

Due to the relationship between visual, motor, and cognitive functions and driving ability, the following commonly used and well-known tests in daily geriatric practice were selected. The following is a brief introduction to each test and its relationship with driving performance.

#### 2.1.1. Visual Function

Visual acuity and contrast sensitivity were used to assess visual function. Visual acuity was assessed by the Snellen Chart, scoring from 0 to 1.2. Peripheral vision is reduced with increasing age [23], which can result in difficulty in detecting relevant traffic stimuli in the driver’s periphery.

Contrast sensitivity was measured by the Pelli–Robson chart [24], whose score ranges from 0 to 2.25. It has been found that normal aging can lead to structural changes in the eye, resulting in reduced visual acuity and contrast sensitivity [9]. This results in increased glare sensitivity, hence increased risk, for the elderly during night driving.

#### 2.1.2. Motor Function

Two tests were selected in this respect. One was the timed get-up-and-go, which helps in analyzing locomotion and mobility [25]. Participants were asked to get up from a chair, walk for 3 m and return, then sit down again. The completion time was recorded as the result measure. A score larger or equal to 14 s shows a high risk of falling and instability. Impaired performance is regarded as predictive of slower emergency maneuvers because of prolonged braking time.

The second test is called the functional reach test, which evaluates the physical balance [26]. Participants were required to stand up straight with a single arm extended forward as far as possible without any support. The difference between arm length and the farthest reach attained by the participant is recorded. The rate of falling due to loss of physical balance is found to be closely associated with accident involvement in older drivers [27].

#### 2.1.3. Cognitive Function

Multiple tests were carried out to assess older drivers’ cognitive function.

Mini-Mental State Examination (MMSE): To measure global cognitive functioning in elderly drivers, MMSE is widely used. A lower MMSE score shows impaired cognitive performance. Its value ranges from 0 to 30.

Clock drawing test: This is an alternative screening tool to the MMSE. In this test the participant is given a pre-drawn circle on paper and is asked to draw a clock showing the time “11:10”. Its score ranges from 0 to 7, with lower scores indicating worse performance.

Amsterdamse Dementie Screening (ADS)—Eight Word subtest: This is an auditory verbal learning test assessing memory. First, eight words are delivered to participants orally, and they are asked to recall as many as possible; repetition of this procedure five times results in a number of correct recalls ranging from 0 to 40. After 15 min, participants are asked to recall as many as possible of the 8 words again (0–8). Finally, 8 new words are added to the list, and participants are asked to judge whether each of the 16 words have been tested before (0–16). The results are recorded respectively.

The Rey Complex Figure Test: In this test, participants are asked to first copy the image according to an example, then draw again according to impression after the example is removed, and finally draw again by memory 15 min later. The three drawings are scored separately, with a value ranging from 0 to 36. It has been found that this test is predictive of driving errors among elderly drivers [28].

WAIS III—Digit Span: This test is used to examine working memory capability. Participants are asked to repeat a progressing number of digits which are read by an experimenter in a sequence. The test consists of two parts. In part 1 the participants recall in the same order as in the presentation, and in part 2 the order is reversed. The maximum number of digits recalled is recorded. It has been found that the older drivers’ working memory performance is associated with their on-road driving abilities [29].

Trail Making Test (TMT): This test consists of part A and B, both of which are widely used in the field of fitness-to-drive. Trail A consists of 25 numbered dots and participants have to search and tie successive numbers on a screen or sheet of paper. In part B, the participants have to do the same alternatively with numbers and letters in alphabetical order (1–A–2–B). TMT A predominantly measures visual search and working memory, whereas TMT B measures attention shifting in addition to increased demands on working memory. Research has found that TMT, particularly the TMT B score minus the TMT A score, is predictive of crashes [30].

Useful Field of View (UFOV): This consists of three subsets that assess processing speed, divided attention, and selective attention. The response time is recorded in milliseconds, with the value ranging from 16.7 to 500 ms. The longer the response time, the worse the performance in the test. It has been found that UFOV is a strong predictor of both retrospective and prospective crashes in the general population of older adults [31].

Stroke Drivers Screening Assessment—Knowledge of Road Signs: In this test, participants are presented with 20 pictures of traffic signs, and are asked to match the signs to the presented traffic situation. Two points are given for a correct match within 3 min and 1 point is given for a correct matching beyond 3 min.

Proteus Maze test: This test examines the ability to use planning, patience, and mental alertness in a novel and concrete performance task. Participants are required to start in the middle of a paper maze and trail through it until they reach the exit without crossing lines or lifting their pencil from the paper. A total of 10 mazes are presented with increasing complexity, and the index number of the most complicated maze completed is recorded. Performance in this test has been found to be significantly related to driving ability ratings [32].

### 2.2. Simulated Driving Test

The STISIM Drive v3 Vehicle Driving Simulator (Systems Technology Inc. Hawthorne, California, USA) with 180° surround projection and field of view was used to carry out the simulator driving experiments. After two practice drives for participants to get used to the simulated driving environment, the test was conducted in 3 different scenarios (i.e., urban, rural, and motorway) at 2 levels of complexity (i.e., high and low traffic flow). The data acquisition frequency was 60 Hz. Participants experiencing simulator sickness were excluded from the test. This test was performed in two different ways:

#### 2.2.1. Observation-Based Assessment

The participants’ driving ability was rated by observation. A test ride to investigate practical fitness-to-drive—Belgium version (TRIP) [33] with 11 subscales focusing on various aspects of driving behavior was completed by the experimenter. Each was scored on a four-point scale, with a total score range of 0–44. The assessment does not need any information collected from the driving simulator, which means that a simplified driving simulator without data collection function is sufficient for this test.

#### 2.2.2. Performance-Based Assessment

The data collected from the simulator were used to measure the driving ability of older adults. Average speed and standard deviation of lateral position (SDLP) were used as performance parameters in the normal driving section. In addition, the simulated driving scenes also included some specific events that are difficult for older drivers, and participants’ performance on these scenes were collected. The detailed description of these events are as follows.

Merging into traffic: The participants were supposed to start from an on-ramp of the motorway and to merge into the passing traffic. The distance traveled (in meters) when they completed the merging and lane changing operation was recorded.

Maximum deceleration-stop sign: The maximum deceleration (in m/s^2^) at the point 100 m before a stop sign-controlled intersection that the participants approached in a rural area was recorded. This was done to measure abruptness of breaking and anticipation.

Initial brake point-pedestrian crosswalk: The participants drove along a rural road and encountered a pedestrian at the crosswalk ahead. It was preset that the pedestrian started to cross the road when the time to collision (TTC) was 3 s. The distance (m) from the crosswalk when the participant started to brake was recorded.

Turning left-gap acceptance: A traffic flow on the intersecting road was presented with the headway increasing by seconds when the participants were asked to turn left. To avoid simulator sickness, participants did not have to actually make a left turn, but simply indicated the gap they decided to cross. The length of the chosen gap (s) was recorded.

Detection time to road hazard: The participant encountered an unexpectedly crossing pedestrian on a rural road, and had to brake hard to avoid a collision. The detection time was recorded as the time from the pedestrian started to cross to the participant’s first release of the throttle (10% released).

Reaction time to road hazards: Similar to the detection time to hazard, reaction time was recorded as the time in seconds from hazard to the first input of the brake pedal. The road hazard in a high traffic flow scene was signaled with a precursor (a bus stop), whereas in low traffic flow scene it was not.

### 2.3. On-Road Driving Test

The on-road driving test was conducted according to Belgium’s official license issuing procedure, with the presence of a specialized fitness-to-drive evaluator from Center for Determination of Fitness to Drive and Car Adaptations (CARA). Based on observation, the assessor scored the driving performance of each participant by TRIP assessment and classified them as "fit" or “unfit” to drive.

## 3. Methodology

In this study, the fitness-to-drive assessment models were constructed with the variables selected from the results of the functional ability test and the simulated driving test as inputs and the outcome of the on-road driving test as the output. In this respect, different assessment combinations were considered, and various modeling techniques were applied. The generalization ability of each model was also evaluated by “hold-out”.

### 3.1. Variable Selection and Assessment Combinations

To perform an effective fitness-to-drive assessment of older drivers, the most valuable predictive set with appropriate number of variables should be extracted. The selection is based on statistical significance and effect size of the correlation with the pass/fail outcome of the on-road driving test. In terms of the Pearson correlation coefficient, the Cohen’s criterion for small, medium, and large effect sizes are 0.1, 0.3, and 0.5, respectively [34]. In this study, variables with an effect size ≥0.3 (more than medium correlation) and significant at the 0.01 level were selected.

In the modeling section, depending on the category of the assessment each test belongs to, 4 types of assessment combinations were considered in this study:

Alternative I—assessing fitness-to-drive by the functional ability test only;

Alternative II—assessing fitness-to-drive by the functional ability test and the simulated driving test (observation based);

Alternative III—assessing fitness-to-drive by the functional ability test and the simulated driving test (performance based); and

Alternative IV—assessing fitness-to-drive by all tests.

The main reason for considering these four alternatives for fitness-to-drive assessment of older drivers is that the cost (including labor, raw material, and time) of these assessment methods increases gradually. Alternative I is used to evaluate the effect of the functional ability test alone on the fitness-to-drive assessment, which requires only several clinical screening tools, and thus costs the least. Then, the observation-based simulator driving test is included in the test battery, and requires a simplified driving simulator and an experimenter to rate the performance by observation. Alternative II is therefore able to evaluate the contribution of observation-based simulator driving to the fitness-to-drive assessment. Next, the performance-based simulator driving test is combined with the functional assessment, in which a more sophisticated device is applied to collect the performance parameters from simulated driving. By comparing the results from Alternative III with those from Alternative II, a more effective method with respect to the simulated driving assessment can be determined. Finally, Alternative IV considers the results from all tests, which also implies the highest cost.

### 3.2. Modeling Techniques

The fitness-to-drive assessment modeling process is essentially a binary classification problem. Thus, in contrast to the traditional statistical analysis applied in previous studies, advanced data mining and machine learning techniques, such as random forest (RF), support vector machine (SVM), and gradient boosted machine (GBM), are investigated in this study. First, we briefly introduce the principles and characteristics of these three techniques.

The random forest is a supervised learning algorithm which builds multiple decision trees and merges them together for a more accurate prediction. It combines the idea of the random subspace method and bagging [35,36]. The decision trees in the random forest use information gain to split a node, which is calculated based on the Gini index or entropy. This method can generally avoid the problem of overfitting, and is able to handle a large number of input variables. Moreover, feature importance can be evaluated by mean decreased accuracy and mean decreased Gini in “out of bag” (OOB) data.

The support vector machine has been increasingly used in traffic safety research, such as in studies of traffic incident detection, driver classification, and crash severity [37,38]. The principle of the SVM is to find a hyperplane to separate two types of points, to maximize the margin between points closest to both sides of the hyperplane; points on the margin are defined as support vectors [39]. In linear inseparable cases, the input vectors can be mapped to a high-dimensional feature space, in which the samples can be linearly separated. To avoid the complex calculation of the inner product in high-dimensional feature space, the concept of the kernel function is introduced, including polynomial kernel, Gaussian kernel (radial basis function kernel), and sigmoid kernel. Overfitting can be prevented by applying soft a margin.

The gradient boosted machine is an extremely powerful machine learning algorithm that removes the weak effect of all of the trees and results in a powerful “committee” of trees by minimizing the error, with high flexibility for parameter tuning [40]. The idea of gradient boosting is that in a suitable cost function, boosting can be interpreted as an optimization function. Gradient boosting minimizes the mean squared error (MSE) loss function and the mean absolute error. This can be considered a gradient descent algorithm, which is a generic optimization algorithm capable of finding optimal solutions. A GBM can also determine the importance of variables.

### 3.3. Model Evaluation

To evaluate the generalization ability of the models, the dataset was divided into training and testing sets with the ratio of 0.7:0.3. Moreover, the quantity imbalance between fit and unfit older drivers (61:31) was considered by applying the Synthetic Minority Oversampling Technique (SMOTE) in R studio (R Core Team (2020). R: A language and environment for statistical computing. R Foundation for Statistical Computing, Vienna, Austria). In contrast to the random oversampling algorithm, the basic idea of SMOTE is to analyze the minority samples and synthesize new ones to add to the dataset according to the minority samples.

In this study, the original training data was processed by the SMOTE algorithm before the model was constructed to improve the model accuracy. All models (3 modeling techniques with 4 types of assessment combinations, i.e., 12 models in total) were built in R studio. The parameters of each model were tuned respectively by cross validation.

Overall accuracy, sensitivity, specificity, positive predicted value, negative predicted value, and the area under the receiver operating characteristic (ROC) curve (AUC, if applicable) were used for evaluating the model performance on the original test set. The calculation of the above indicators is shown in the following equations:

(1) $$\mathrm{Sentivity}\;=\;\frac{a}{a+c}$$

(2) $$\mathrm{Specificity}\;=\;\frac{d}{b+d}$$

(3) $$\mathrm{Positive}\;\mathrm{predicted}\;\mathrm{value}\;=\;\frac{a}{a+b}$$

(4) $$\mathrm{Negative}\;\mathrm{predicted}\;\mathrm{value}\;=\;\frac{d}{c+d}$$

where “a” represents the number of participants who are assessed as fit-to-drive by both the model and the on-road driving test, “b” represents the number of participants who are assessed as fit-to-drive by the model but actually unfit, “c” represents the number of participants who are assessed as unfit-to-drive by the model but actually fit, and “d” represents the number of participants who are assessed as unfit-to-drive by both the model and the on-road driving test.

## 4. Application and Results

In this study, a complete dataset of 92 participants with 34 relevant influencing factors were used for analysis. The remaining 44 participants were excluded because of dropping out, simulator sickness, unfinished on-road driving test, or missing data. The average age of the sample is 78.22 years old, with 55.45 years of driving experience on average. A share of 59.8% of the participants drive at a daily frequency, and 98.9% drive more than once a week. Moreover, 67.4% of the sample drive more than 5000 km per year, and 33.7% drive more than 10,000 km per year. According to the results of the on-road driving test, 61 older drivers are considered as fit-to-drive, whereas the remaining 31, who failed the test, are now regarded as unfit-to-drive.

### 4.1. Descriptive Statistics and Variable Selection

The descriptive statistics of the functional ability test and their correlation coefficients with the on-road driving test are presented in Table 1. Similarly, results from the simulated driving tests are shown in Table 2.

According to the variable selection criteria, 8 of the 34 influencing factors were selected. However, “UFOV—divided attention” and “UFOV—selective attention” were highly correlated (r = 0.901, *p* = 0.000). To avoid multicollinearity, the factor with the lowest effect size was excluded. Consequently, seven influencing factors in total were selected to assess the fitness-to-drive for the elderly.

Amongst others, visual acuity, functional reach, UFOV—selective attention, and knowledge of road signs belong to the functional ability test. Merging into traffic and road hazard reaction time—without precursor are parameters from the performance-based simulator driving test. Additionally, the TRIP score is the measure of observation-based simulator driving assessment. All of these are closely correlated with the on-road driving test outcome and are statistically significant.

### 4.2. Model Evaluation

The input variables of the models were chosen from the seven influencing factors selected above. Considering different types of assessment combinations, 4 alternatives were developed, as shown in Table 3. The evaluation results based on different modeling techniques are presented in the following sections.

#### 4.2.1. Assessing Fitness-to-Drive by Functional Abilities Test Alone

- RF-I

The prediction accuracy of this model was 71.5%, with sensitivity of 0.81, specificity of 0.58, positive predicted value of 0.72, and negative predicted value of 0.70. The AUC is 0.71. The number of trees (ntree) and the number of variables randomly selected at each split (mtry) in RF were tuned as 500 and 2, respectively, for best prediction, and these parameters were held consistent for all RF models.

- SVM-I

The model correctly predicted 81% of the older drivers who are fit-to-drive and 58% who are unfit-to-drive, with the positive and negative predicted values of 0.72 and 0.70, respectively, resulting in an overall accuracy of 71.5%. The ROC curve was not applicable for SVM because there is no threshold for classification that can be adjusted.

- GBM-I

The GBM model correctly classified 76% of the participants who are fit-to-drive and 71% unfit, with an overall accuracy of 75%. The area under the curve was 0.84 (sensitivity = 0.76, specificity = 0.71, positive predicted value = 0.88, negative predicted value = 0.50).

In summary, the results show that the three models above based on the functional ability test alone cannot achieve both high sensitivity and specificity. In particular, the recognition ability of unfit-to-drive older drivers is not adequate. Among others, the GBM model has the highest overall accuracy, specificity, and AUC value, while its sensitivity score is slightly lower than those of the other two models, which means that it is relatively more likely to misclassify fit-to-drive as unfit.

#### 4.2.2. Assessing Fitness-to-Drive by Functional Abilities Test and Simulated Driving Test (Observation-Based)

- RF-II

The outcome measure of observation-based simulator driving test, i.e., the TRIP score, was added to the assessment. The RF model correctly predicted 82% of the fit-to-drive participants as fit and 63% of unfit-to-drive participants as unfit (sensitivity = 0.82, specificity = 0.63, positive predicted value = 0.77, negative predicted value = 0.70), resulting in a predictive accuracy of 75% and an AUC value of 0.72. Compared with RF-I, all evaluation indicators of RF-II were slightly improved, with the exception of the negative predictive value which remained unchanged.

- SVM-II

The results of the prediction show that 83% of the participants who were predicted as fit-to-drive actually passed the on-road driving test, and that 60% of the participants predicted as unfit-to-drive actually failed in the on-road driving test (sensitivity = 0.79, specificity = 0.66, positive predicted value = 0.83, negative predicted value = 0.60). The overall prediction accuracy of the model was 75%, which was improved relative to SVM-I.

- GBM-II

This model correctly classified 80% of the fit-to-drive older adults as fit and 75% of those unfit-to-drive as unfit (sensitivity = 0.80, specificity = 0.75, positive predicted value = 0.89, negative predicted value = 0.60), with an overall accuracy of 78.6% and an AUC value of 0.85. Similar to the case in RF-II, the performance of GBM-II was better than that of GBM-I in all aspects.

From the results above, including observation-based simulator driving test in the assessment was proven to be beneficial for predicting fitness-to-drive of the elderly. Among the three techniques discussed above, RF had the highest sensitivity and the lowest specificity, SVM had the lowest specificity, and GBM still had the highest overall accuracy, specificity, and AUC value.

#### 4.2.3. Assessing Fitness-to-Drive by Functional Abilities Test and Simulated Driving Test (Performance-Based)

- RF-III

The observation-based simulator driving test was replaced by performance parameters collected from simulated driving, i.e., "Merging into traffic” and “Road hazard reaction time—without precursor”. For this set of variables, the RF model correctly classified 83% of the fit-to-drive participants as fit and 70% of the unfit-to-drive participants as unfit (sensitivity = 0.83, specificity = 0.70, positive predicted value = 0.83, negative predicted value = 0.79), resulting in an overall accuracy of 78.6%, and the AUC value of this model was found to be 0.76. The performance of RF-III was better than that of RF-II considering all evaluation indicators.

- SVM-III

The replacement also increased the predictive performance of SVM to a certain extent. This model correctly classified 77% of the participants who pass the on-road driving test as fit and 83% of participants who fail as unfit (sensitivity = 0.77, specificity = 0.83, positive predicted value = 0.94, negative predicted value = 0.50). The overall accuracy was 78.6%.

- GBM-III

The overall accuracy of this model was 82.2%, with sensitivity of 0.84, specificity of 0.78, positive predicted value of 0.88, negative predicted value of 0.70, and AUC value of 0.86. After replacing observation-based indicators by performance-based indicators for assessment, the performance of GBM-III was slightly improved comparing to GBM-II.

The results above demonstrate that the performance indicators from simulated driving test were more effective in assessing fitness-to-drive for the elderly than the observation-based indicators. In this assessment method, SVM had the highest specificity, which means that its misclassification probability of unfit-to-drive was the lowest. Regarding for the other model evaluation indicators, GBM achieved the highest scores.

#### 4.2.4. Assessing Fitness-to-Drive by All Tests

- RF-IV

After combining all types of tests, the overall accuracy of the RF model was improved from 78.6% (RF-III) to 82.2%. The model correctly classified 88% of the fit-to-drive participants and 72% of the unfit-to-drive participants, respectively (positive predicted value = 0.83, negative predicted value = 0.80), reaching an AUC value of 0.85.

- SVM-IV

This model performed even better than RF-IV, with an overall accuracy of 85.7% (sensitivity = 0.85, specificity = 0.87, positive predicted value = 0.74, negative predicted value = 0.90), and could suitably identify older adults who were unfit to drive.

- GBM-IV

This model correctly classified 94% of the fit-to-drive participants and 90% of the unfit-to-drive participants (sensitivity = 0.94, specificity = 0.90, positive predicted value = 0.94, negative predicted value = 0.90), reaching the highest accuracy of 92.8% and the highest AUC value of 0.97 among all of the 12 models, in addition to the highest sensitivity and specificity.

**Figure 1:**
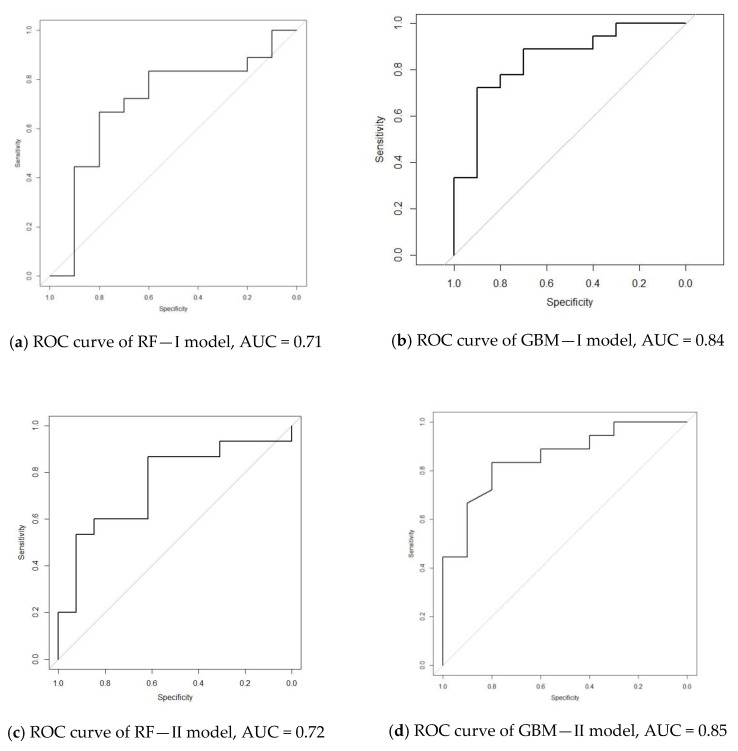

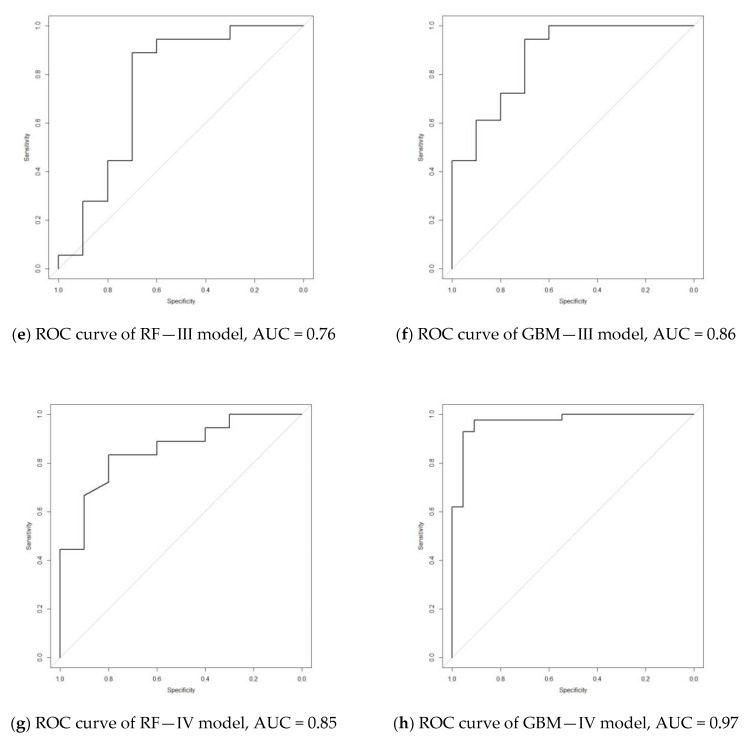
ROC curves of each model. The horizontal axis represents specificity (from 1 to 0), and the vertical axis represents sensitivity (from 0 to 1).

Figure 1 presents the ROC curves of all the models.

Table 4 provides a summary of the performances of the 12 models discussed above. In cross-sectional comparison, the GBM model was found to be better than RF and SVM in overall accuracy regardless of the type of assessment or combination of assessments used for prediction, whereas the indicators of sensitivity and specificity fluctuated among these three methods. Nevertheless, irrespective of the method applied, the prediction accuracy of the models reached the highest when combining all tests, i.e., Alternative IV. To summarize, GBM-IV was the best model among all of the 12 models, with sensitivity and specificity over 0.9, and AUC value close to 1.

**Figure 2:**
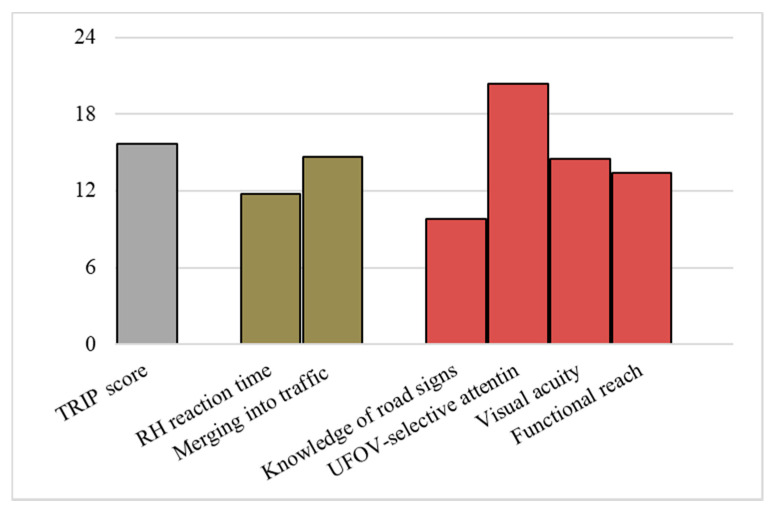
Relative importance of selected variables in the GBM model.

To determine which variable (s) significantly influence the performance of the model, all of the variables were ranked by the feature importance algorithm in GBM. As shown in Figure 2, this model takes “UFOV—selective attention” from the functional ability test as the most important influencing factor, followed by the observational “TRIP score”. Regarding driving simulator performance measures, merging distance on a highway is a more important factor.

## 5. Discussion

### 5.1. Principal Findings

In this study, assessing fitness-to-drive for the elderly based on different types of tests was explored to identify the most effective alternative to on-road driving test. In total, seven variables from either the functional ability test or the simulated driving test were selected given their significant correlation with the on-road driving test outcome. Three machine learning techniques and four test combinations were considered as potential alternatives. As a result, the GBM model using all variables was found to be the best option, with overall accuracy of 92.8%. The results of feature importance suggest that “UFOV—selective attention” is the most important influencing factor, followed by the observational “TRIP-score” and “Merging into traffic” in simulated driving.

### 5.2. Influencing Factors for Fitness-to-Drive

Driving is a complex task that can be affected by many factors, including the visual, motor, and cognitive conditions of a person, and requires competent functional ability for safe control. A good driver needs to be able to visually interpret traffic and road conditions, have sufficient knowledge of road signs, and be physically stable and active to control the vehicle. Literature on age-related driving behavior studies is vast, but age alone is not a good indicator of driving performance and skills, because many physical changes can occur during aging, resulting in impaired functional ability and driving ability. In this study, only a weak association between age and on-road driving test outcome was found (*p* = 0.02, r= −0.21). Therefore, age was not included in the assessment. This result is consistent with the conclusion drawn in many previous studies [7,8].

Among factors selected from the functional ability test, visual acuity is crucial to driving safety. A lower visual acuity is representative of impaired vision and hence poor driving, which can also influence older drivers’ ability to notice and comprehend the meanings of road signs, thereby putting them at greater risk comparing to younger drivers [41]. Road signs and signals convey important information for driving. Ignoring and misunderstanding traffic signs have a negative impact on safety and efficiency of traffic. A higher score on knowledge of road signs also indicates a better executive function. The attention required for driving is also valued, which is measured by the UFOV test. It has been found that the “UFOV—selective attention” is the best predictor of driving performance in complex situations among the subtests of UFOV [31] and, in the current study, “UFOV—selective attention” was not only selected but also identified as the most important factor among all factors in the GBM model. In addition, the results indicate that the physical balance measured by the functional reach test is also important for safe driving of the elderly, which was found to be strongly associated with poor driving skills and accident involvement in older drivers [27].

The factors selected from driving simulator tests indicate that road hazards’ perception can distinguish older adults as safe or unsafe drivers. Older drivers have been found to be slower at responding to hazards and traffic conflicts due to cognitive and visual impairments [42]. In addition, unfit-to-drive older drivers suffer from reduced information processing speed and perception ability, thus taking larger distance and more time to merge into traffic flow. The selection of the variable “merging into traffic” is supported by the findings of previous studies that older drivers usually have trouble quickly moving into traffic streams [43]. In addition to the performance variables mentioned above, the observation-based TRIP score is also considered important for fitness-to-drive assessment. The TRIP score results from the simulated driving test, and the high correlation between this score and the pass/fail outcome of the on-road driving test also justifies the effectiveness of simulation.

### 5.3. Fitness-to-Drive Assessment Modeling

Choosing a fitness-to-drive assessment model or a classifier is critical, especially when dealing with imbalanced classes. In most cases, classifiers can only perform well at predicting the major class, but result in more misclassifications of the minor classes. To address this problem, the data were balanced by the SMOTE algorithm. In contrast to previous studies that did not conduct model validation, in the current study, 70% of the data was used for training and the remaining 30% for evaluating the generalization error of the model. Compared with RF and SVM, the GBM model proved to be the best option. This is attributed to the greedy nature of GBM, which minimizes the information loss function of all features and constructs successive training sets based on incorrectly classified examples.

The performance of the models initially constructed by the functional ability test alone is not satisfactory, which supports the previous viewpoint that it is difficult to identify the fitness-to-drive among the elderly merely by functional assessment [11]. When considering different combination of the tests, the percentage of correctly classified drivers increases significantly when performance variables collected from the simulator driving test are included. Adding the observation-based TRIP score from the simulator driving test can also improve the performance of the model, but the effect is not as great as performance-based assessment, despite its strong correlation with the on-road driving test outcome. Finally, a combination of all seven variables from all of the tests increased the predictive accuracy from 78.5% to 82.2%, 75% to 85.7%, and 82.2% to 92.8% in RF, SVM and GBM, respectively.

In summary, we conclude based on this study that the best alternative for fitness-to-drive assessment among older drivers is the that which combines all variables from both the functional ability test and the simulated driving test, with the model trained via the gradient boosted machine, because it results in the highest predictive accuracy of 92.8% and the largest AUC of 0.97. Moreover, such a model is recognized by its capability to well predict both fit and unfit older drivers, thus decreasing the chance of misclassification of the minor class. However, it should be noted that to perform such an assessment in practice, the highest cost regarding labor, raw material, and time is also required.

### 5.4. Limitations

We believe that the results obtained from this study are valuable from both theoretical and practical perspectives. However, some limitations in terms of experiment design and model development are present and in need of further investigation.

First, the scores from the on-road driving test are used as a criterion to distinguish between fit- and unfit-to-drive older adults, and for different models’ performance evaluation and comparison. Although the on-road driving test is generally regarded as the “gold standard” for fitness-to-drive assessment, in reality uncertainty exists about the extent to which the licensing procedure reflects the true level of driving ability among older adults. In addition, in this study, fit- or unfit-to-drive were the only two outcomes from the on-road driving test. However, individuals may lie between the absolute categories of “fit” and “unfit”, who could be treated as safe drivers under certain conditions. This situation was not considered in the current research.

Regarding the simulated driving experiments, a gap clearly exists between simulation and naturalistic driving, regardless of the improvements made to increase the authenticity of the former [44]. For instance, in the “maximum deceleration” and “initial break point” events, the simulation is not closely indicative of real-world braking due to the lack of suitable feedback cues. Additionally, maintaining a minimum highway speed is not included in the performance-based simulator assessment, which might be an important variable to be considered. For instance, drivers who drive faster would brake harder when they encounter a crossing pedestrian. Furthermore, even if the reaction time of individuals is the same, a higher speed will lead to a longer distance traveled before braking, thus affecting the performance in the “initial brake point” event.

Moreover, based on a literature review [19,34], variables with effect size greater than 0.3 were selected for further analysis. Such a rule is feasible in practice but still lacks well-grounded theoretical support. Other criteria might be considered in the future and a sensitivity analysis should be conducted.

In addition, 10% of unfit-to-drive older adults were not discovered by the GBM-IV model, and 6% of capable older drivers were suggested to cease driving by this model; that is, there is still room for model improvement. For instance, other potential factors should be explored, such as the measurement of risk-taking tendencies and aggressive driving behaviors [45]. To achieve this goal, appropriate indicators need to be first developed and refined.

## 6. Conclusions and Future Research

Conflict between the mobility needs of older drivers and their impaired driving ability is a long-standing issue. Older drivers may drive beyond their fitness limits at the expense of public safety. Conversely, safe drivers may be forced to cease driving. Therefore, there is an urgent need to establish a fitness-to-drive assessment method to distinguish unsafe from safe older drivers.

By combining a functional ability test, and observation-based and performance-based simulator driving tests, in different ways, four potential alternatives to the on-road driving test were investigated and further compared in this study. The best approach combined all seven variables from both the functional ability test and the simulated driving tests, with the model trained using a gradient boosted machine, which correctly classified 94% of safe older drivers and 90% of unsafe older drivers, with overall accuracy of 92.8%. Moreover, visual acuity, functional reach test, UFOV—selective attention, knowledge of road signs, merging into traffic, reaction time to road hazard—without precursor, and TRIP score were selected as the most relevant variables for the fitness-to-drive assessment, in which UFOV—selective attention was considered to be the most important influencing factor, followed by the observational TRIP score and merging into traffic in simulated driving.

It should be noted that although the motivation of this study is to develop a fitness-to-drive assessment method as an alternative to the on-road driving test, “replacing” it completely by applying either the functional ability test, the simulated driving test, or both together remains a distant goal. The on-road driving test remains the “gold standard” for fitness-to-drive assessment currently. However, the results from this study are encouraging and promising, and offer a valuable reference for some older drivers to either adjust their driving behavior, apply auxiliary devices, or cease driving. Although not perfect, it provides an opportunity to rethink the traditional method used for fitness-to-drive assessment.

In the future, other potential influencing factors, such as risk-taking tendencies, aggressive driving behaviors, and maintaining a minimum highway speed, should be investigated in simulator driving tests. Moreover, to guarantee the convenience and life quality of the elderly, in addition to the safety of the public, further research can focus on driving ability training approaches and the design of an intelligent auxiliary driving system for unfit-to-drive older drivers.

## Figures and Tables

**Table 1:** Descriptive statistics and correlation coefficients of the functional test measures.

	Mean	SD	Sig.	r
Visual acuity	0.71	0.18	0.001 *	0.30 ^1^
Contrast sensitivity	1.78	0.2	0.016	0.22
Timed get up and go	9.5	3.2	0.009 *	−0.24
Functional reach	32	6.5	0.000 *	0.32 ^1^
MMSE	26.9	2.3	0.107	0.15
Clock drawing test	4.9	1.3	0.151	0.13
ADS eight-word test—direct recall	29.0	6.8	0.008 *	0.24
ADS eight-word test—delayed	4.8	2.2	0.438	0.07
ADS eight-word test—recognition	15	1.6	0.011	0.23
RCFT—Copy Trail	27.8	4	0.095	0.15
RCFT—Immediate Recall	15	5.9	0.004 *	0.26
RCFT—delayed recall	14.4	5.7	0.042	0.22
WAIS digit span—forward	5	0.8	0.012	0.23
WAIS digit span—backward	3.8	1	0.107	0.15
Trail making test—A	59.6	29.7	0.013	−0.23
Trail making test—B	140.7	52.8	0.006 *	−0.28
UFOV—processing	58.3	83.4	0.008 *	−0.24
UFOV—divided attention	190.6	163.9	0.001 *	−0.30 ^1^
UFOV—selective attention	247.2	146.3	0.000 *	−0.32 ^1^
Knowledge of road signs	14	5.7	0.000 *	0.36 ^1^
Proteus Maze	9.5	2.3	0.113	0.15

* *p* < 0.01, ^1^ effect size ≥ 0.3.

**Table 2:** Descriptive statistics and correlation coefficients of the simulated driving measures.

	Mean	SD	Sig.	r
Average driving speed—urban	50.2	6.3	0.350	−0.10
Average driving speed—rural	63.9	9.3	0.762	0.03
Standard deviation of lateral position—urban	0.24	0.06	0.194	−0.14
Standard deviation of lateral position—rural	0.22	0.07	0.274	−0.12
Merging into traffic—distance	917.9	126.3	0.008 *	−0.31 ^1^
Maximum deceleration—at stop sign	−6.5	1.9	0.408	0.10
Initial brake point—at zebra crossing	45.7	14.5	0.643	0.05
Turning left—gap acceptance	7	1.6	0.096	0.20
Road hazard detection time—without precursor	0.92	0.68	0.020	−0.26
Road hazard detection time—with precursor	1.0	1.2	0.379	0.10
Road hazard reaction time—without precursor	0.21	3.6	0.006 *	−0.30 ^1^
Road hazard reaction time—with precursor	0.10	9.4	0.822	0.02
TRIP score (observation based)	35.5	6.0	0.002 *	0.326 ^1^

* *p* < 0.01, ^1^ effect size ≥ 0.3.

**Table 3:** Different variables used in the designated alternatives.

**Alternative I—Fitness-To-Drive Assessment Using Functional Abilities Test Alone**
Functional reach
Knowledge of road signs
UFOV—selective attention
Visual acuity
**Alternative II—Fitness-to-drive assessment using functional abilities test and simulated driving test (observation-based)**
Functional reach
Knowledge of road signs
UFOV—selective attention
Visual acuity
TRIP score
**Alternative III—Fitness-to-drive assessment using functional abilities test and simulated driving test (performance-based)**
Functional reach
Knowledge of road signs
UFOV—selective attention
Visual acuity
Merging into traffic
Road hazard reaction time—without precursor
**Alternative IV—Fitness-to-drive assessment by combining all tests**
Functional reach
Knowledge of road signs
UFOV—selective attention
Visual acuity
Merging into traffic
Road hazard reaction time—without precursor
TRIP score

**Table 4:** Summary of model performances.

	Accuracy (%)	Sensitivity	Specificity	AUC
RF—I	71.5	0.81	0.58	0.71
RF—II	75.0	0.82	0.63	0.72
RF—III	78.6	0.83	0.70	0.76
RF—IV	82.2	0.88	0.72	0.85
SVM—I	71.5	0.81	0.58	-
SVM—II	75.0	0.79	0.66	-
SVM—III	78.6	0.77	0.83	-
SVM—IV	85.7	0.85	0.87	-
GBM—I	75.0	0.76	0.71	0.84
GBM—II	78.6	0.80	0.75	0.85
GBM—III	82.2	0.84	0.78	0.86
GBM—IV	92.8	0.94	0.90	0.97

-AUC is not applicable for SVM.
